# The Texas Medical College and Redivivus

**Published:** 1887-07

**Authors:** 


### EDITORIAL DEPARTMENT.

F. E. DANIEL, M. D., EDITOR AND PUBLISHER.

COLLABORATORS:

*E. J. Doering, M. D., Chicago.*

*Geo. Cupples, M. D., San Antonio.*

*Odo Betz, M. D., Germany.*

*E. J. Beall, M. D., Fort Worth, Texas.*

*T. C. Osborn, M. D., Cleburne, Texas.*

*Wm. Penny, M. D., New York.*

*H. O. Marcy, M. D., Boston.*

*C. K. Gregg, M. D., Mexico.*

*R. M. Swearingen, M. D., Austin.*

*C. H. Wilkinson, M. D., Galveston.*

*J. W. McLaughlin, M. D., Austin.*

*R. H. L. Bibb, M. D., Mexico.*

### THE TEXAS MEDICAL COLLEGE AND HOSPITAL, REDIVIVUS.

We think it can be shown that, looked at from an economical standpoint, at least, a medical college in Texas has become a necessity. We will not discuss the question of "too many medical colleges;" there are too many *elsewhere*—and—one too few in Texas; and as it is proposed to have one of the highest grade, it would not come within the general objection.

The population of the State in 1881 (last census) was 1,500,000, and the number of practicing physicians was put down at 3003. The number of students who left the State and attended lectures at the various medical colleges in the United States was then 257, all told. It is claimed now, that the population is 2,700,000, an increase of four-fifths of the whole number, or 80 per cent, in six years. This ratio, applied to the medical students, would give Texas now 462, annually; and as the population is said to be steadily increasing at the rate of a quarter million each year, the number of students can be easily estimated, Suffice it say, that perhaps with the exception of Missouri, which has fourteen medical schools, Texas furnishes the largest number of students of any State in the union. These go out of the State to find that education which it should be the pride and boast of the State to be able to furnish, and not to furnish which, is a reproach on our boasted civilization and our large school fund. It is a mere matter of calculation how much money is thus drawn from the State; an immense amount.

Recognizing and appreciating this necessity, the physicians of Galveston have been moving in the matter, and we are pleased to be able to state on the best authority, that a medical college in that city may now be regarded as an accomplished fact. But for unforseen circumstances, lectures would have been delivered this fall. This circumstance was the inability of the trustees to get possession of the City Hospital buildings—a lease to which has been given by the council to the medical faculty, or, rather to the Board of Trustees, the lease to run until such time as a medical branch of the State University shall have been established, as provided by law. The builings are, at present, occupied by one of the public schools, and the school trustees refuse to vacate them until they can secure other quarters. This, it is hoped, will be speedily arranged, when, there being no other impediment the Medical College will be put in active operation at once.

It is by no means, a private enterprise, but one in which the entire profession of the State is interested ; and being assured that our readers will be pleased to be informed of what is being done, and has been done, toward the consummation of this cherished object, we give below the facts.

The "TEXAS MEDICAL COLLEGE AND HOSPITAL," has been reorgnized, and a Board of Trustees elected, with Dr. D. F. Stuart, of Houston, for President. Appropriate committees have been selected, and much solid work has been done. Some $20,000 for the purchase of laboratory and general apparatus is guaranteed. The Sealy Hospital is to be speedily erected, (with the fund left for that purpose by the late John Sealy.) The faculty of the college is to be the Medical and Surgical staff of this Hospital. It has been thought best to postpone the final selection and announcement of the faculty, until all details are arranged; but it is an open secret, that certain distinguished physicians of the State will occupy the important chairs. Suffice it to say, that, with a first class college and abundant clinical material, furnished by two large hospitals, it will be the end and aim of those entrusted with the selection of the faculty to choose one which shall be everywhere recognized as men of first class requirements and teaching ability.

It is proper to state that there is an understanding and agreement between the movers in this enterprise and the Regents of the State University, that when the State shall be in position to carry out the law with regard to founding a medical branch of the State University, this organization shall be dissolved, and all property belonging to it, is to be turned over to the Regents for the use of the latter institution. The fund above mentioned, for the purchase of apparatus, has been raised by subscription amongst the wealthy citizens of Galveston, with this understanding. Dr. Wooten, President of Board of Regents, has recently shown that it will be some ten years before the University fund will be in such shape as to enable the Regents to carry out the plan; meantime, by or before the fall of 1888 we will have in full operation, a first class medical college, where the 460 Texas students will find every facility for acquiring as thorough and complete an education as can be had in any medical center of the North, East or West. So mote it be.

